# Job Insecurity: A Comparative Analysis between Migrant and Native Workers in Australia

**DOI:** 10.3390/ijerph16214159

**Published:** 2019-10-28

**Authors:** Xiaomin Liu, Steven J. Bowe, Allison Milner, Lin Li, Lay San Too, Anthony D. LaMontagne

**Affiliations:** 1 Institute for Health Transformation, School of Health & Social Development, Deakin University, Geelong, Victoria 3220, Australia; xiaominl@deakin.edu.au (X.L.); tiffany.too@unimelb.edu.au (L.S.T.); 2 Psychiatric Unit, First Affiliated Hospital of Kunming Medical University, Kunming 650032, China; 3 Deakin Biostatistics Unit, Faculty of Health, Deakin University, Geelong, Victoria 3220, Australia; s.bowe@deakin.edu.au; 4 Centre for Health Equity, Melbourne School of Population and Global Health, The University of Melbourne, Melbourne, Victoria 3010, Australia; allison.milner@unimelb.edu.au; 5 Nigel Gray Fellowship Group, Cancer Council Victoria, Melbourne, Victoria 3004, Australia; Lin.Li@cancervic.org.au

**Keywords:** job stressor, occupational exposure, immigrant, overseas-born, native workers

## Abstract

Job insecurity is a modifiable risk factor for poor health outcomes, and exposure to job insecurity varies by population groups. This study assessed if job insecurity exposure varied by migrant status and if the differences varied by gender, age, educational attainment, and occupational skill level. Data were from wave 14 of the Household Income and Labour Dynamics in Australia Survey. The outcome was job insecurity. Exposure was migrant status defined by (1) the country of birth (COB), (2) the dominant language of the COB, and (3) the number of years since arrival in Australia. Data were analysed using linear regression, adjusting for gender, age, educational attainment, and occupational skill level. These covariates were also analysed as effect modifiers for the migrant status–job insecurity relationships. Migrant workers, especially those from non-English speaking countries (non-ESC-born), experienced higher job insecurity than Australia-born workers; however, these disparities disappeared after 11+ years post-arrival. The migrant status–job insecurity relationships were modified by educational attainment. Unexpectedly, the disparities in job insecurity between non-ESC-born migrants and Australia-born workers increased with increasing educational attainment, and for those most highly educated, the disparities persisted beyond 11 years post-arrival. Our findings suggested that continuing language skill support and discrimination prevention could facilitate migrant integration into the Australian labour market.

## 1. Introduction

Psychosocial job stressors are recognized as modifiable risk factors for poor health outcomes [1,2]. Job insecurity refers to ‘the perceived threat of job loss and the worries related to that threat’ [3], which is one of the most widely studied psychosocial job stressors and has been proven to be associated with adverse effects on a wide range of physical and mental health outcomes [3,4,5]. For example, exposure to job insecurity is associated with poor self-reported health [6], increased physical health complaints [7], elevated risk of diabetes [8], coronary heart disease [9], and high blood pressure [10]. Moreover, experiencing job insecurity decreases general psychological well-being [11], and is also associated with burnout [12], depression [13], anxiety [13], and increased frequency of thoughts of suicide [14,15].

Exposure to job insecurity varies by population groups. Workers with lower socioeconomic status are generally more likely to be exposed to job insecurity [5]. For example, perceived job insecurity is more common among workers with a high school or lower educational level compared to those with higher educational levels [6]. Temporary contract workers report higher job insecurity [16], as do manual labourers and older workers [17]. Some studies have reported that men have higher job insecurity than women [18]. Substantial racial differences in job insecurity have also been reported, as racial minority workers are more likely to experience high levels of job insecurity [5]. For example, Black workers have been reported to have higher job insecurity than non-Black workers in the US [6]. Further, migrant workers have reported exposure to higher job insecurity due to their increased likelihood of being employed in precarious jobs [5].

Migrant workers are a heterogeneous group. Besides the country of birth, the language proficiency of the host country (English in this study) and the number of years since arrival in the host country may have important influences on migrants’ job stressor exposures. Research suggests that language barriers reduce migrants’ opportunities to obtain quality employment [19] and, compared to those with high language proficiency, migrants with low language skill take much longer to obtain their desired jobs [20]. For example, migrants from non-English speaking countries in Australia have been reported to be more likely to work in low-skill positions with more adverse job stressor exposures [21]. Further, migrant workers who have recently arrived may be particularly ‘vulnerable workers’ [22], facing many challenges besides language difficulties, such as unacknowledged foreign credentials and experience [20], acculturation [23], discrimination [24], and lack of social and professional networks [25]. Therefore, they are more likely to be employed in part-time and temporary jobs [26] and involved in so-called ‘survival jobs’ with poor working conditions and increased precarity [27].

Moreover, the interaction of migrant status with gender, age, and socioeconomic status may create more complex patterns of inequalities in job stressor exposures [28] due to so-called ‘double disadvantage’ [29], which arises through the convergence of more than one exposure determinant (also known as intersectionality). Female migrants are more likely to experience combinations of discrimination [30], marginalization in the labour market [31], and lack of power to refuse adverse working conditions [5]; younger and lower educated migrants also experience these disadvantages. Therefore, the disparities in job stressor exposures may be larger between these subgroups of migrant workers and native workers than other subgroups of migrant workers (male, older, and higher educated migrants). For example, the differences in job control between female migrants and Spanish-born workers were reported to be larger than that of male migrants [28]. However, only a very small number of previous studies have compared migrant status–job stressor relationships by migrant subgroups.

The number of migrants in Australia is increasing, and nearly 60% are employed [32]. Despite job insecurity being reportedly common among migrant workers [28,33,34], there are few studies on migrants’ job insecurity in Australia [34,35,36,37]. Among those, Daly, Carey, Darcey, Chih, LaMontagne, Milner, and Reid [37] reported that Australia-born workers had lower job insecurity than migrant workers, and McGuinness and Wooden [35] reported that migrants from non-English speaking countries (non-ESC-born) and recently arrived migrants had higher levels of job insecurity. In this study, we address two research questions: (1) Do exposures to job insecurity differ between migrant workers and native workers? (2) Are ‘migrant status–job insecurity’ relationships modified by gender, age, educational attainment, or occupational skill level? Further, we hypothesise that: (1) migrant workers, especially non-ESC-born and recently arrived migrants, are more likely to experience higher job insecurity than Australia-born workers, and (2) the disparities of job insecurity between migrant and Australia-born workers may be larger for female, younger, and lower educated workers, and workers in low-skill level jobs.

## 2. Materials and Methods

The data were procured from the Household Income and Labour Dynamics in Australia Survey (HILDA). This study was approved by the Deakin University Human Research Ethics Committee (No. 2017-226).

### 2.1. Data Source

HILDA is a nationally representative sample of Australian households, whose data collection began in 2001 and was conducted annually via a stratified three-stage clustered design [38]. In Australia, 488 Census Collection Districts (CDs) were selected; within each CD, 22 to 34 dwellings were selected systematically, and within each dwelling, 1 to 3 households were selected. All household members aged 15 and older were interviewed through face-to-face and telephone interviews. In 2001, there were 13,969 persons who responded to the survey. In 2011, an additional 5451 persons were recruited to top up the sample size to allow a better representation of the Australian population. Wave 14 (2014–2015) was used in the current study, and there were 17,325 individuals who responded to the survey, among which, 16,780 were in the original sample and 545 were new entrants. Socio-demographic characteristics, including migrant status and psychosocial job conditions (insecurity), were available from the HILDA Self-Completion Questionnaire (SCQ), and 15,423 individuals (89.0%) completed the SCQ in wave 14 [39].

### 2.2. Inclusion and Exclusion Criteria

The respondents included in our analysis were aged 15 to 64 years, were employed, and responded to all the items on job insecurity in wave 14. In this wave, 15,231 observations were aged 15–64 years, and of these, 10,575 were employed. Among these respondents, 9043 answered all three items of the job insecurity scale and formed the analytic sample of this study.

### 2.3. Exposure Variables (Migrant Status)

Migrant status was measured in three ways. The first was based on country of birth (COB) only; a binary variable coded as Australia-born versus overseas-born. The second was based on COB and the dominant language of the COB, a three-category variable coded as (1) Australia-born, (2) born in the main-English speaking country (main-ESC-born), and (3) born in the non-English speaking country (non-ESC-born) was generated as a crude proxy for measuring English language proficiency. The third was based on both COB and years since arrival in Australia. A four-category variable including (1) Australia-born, and overseas-born workers of those who (2) arrived ≤5 years ago, (3) arrived 6–10 years ago, and (4) arrived ≥11 years ago. This was generated to provide a crude measure of increasing acculturation over time.

### 2.4. Outcome Variable

We formulated job insecurity using a previously developed scale in HILDA [40]. Job insecurity was measured via three items: “I have a secure future in my job”, “The company I work for will still be in business 5 years from now”, and “I worry about the future of my job”. These were scaled from 1—strongly disagree to 7—strongly agree. The first two items were reversed so that a higher score indicated higher job insecurity. The score of job insecurity was computed by summing the three items running from 3 to 21, with a higher score representing higher job insecurity (Cronbach’s α = 0.67). These items were shown to have good internal consistency in previous Australian studies [15,18,40]. We analysed job insecurity as a continuous measure to optimise discriminatory power.

### 2.5. Covariates

Covariates included gender (binary, i.e., male or female), age (five categories, i.e., 15–24, 25–34, 35–44, 45–54, and 55–64), educational attainment (four categories, i.e., high school or lower, diploma or certificate, bachelor, and postgraduate), and occupational skill level. Based on the Australia and New Zealand Standard Classification of Occupation [41], occupational skill level was categorized into four categories, namely, highest skill (major group 1 and 2: Managers and Professionals), mid–high skill (major group 3 and 4: Technicians and Trade Workers and Community and Personal Service Workers), mid–low skill (major group 5 and 6: Clerical and Administrative Workers and Sales Workers), and lowest skill (major group 7 and 8: Machinery Operators and Drivers and Labourers).

### 2.6. Statistical Analysis

Firstly, we used Chi-squared tests to compare the prevalence of socio-demographic characteristics and occupational skill level by migrant status. Then, descriptive analysis was performed to calculate the mean and standard error of job insecurity by migrant status. Because the HILDA Survey involves a complex design and has unequal probabilities of selection and non-response, Summerfield et al. [42] suggested that these should be taken into account when calculating standard error; sampling weights were used to adjust our prevalence estimates. HILDA User Manual-Release 14 provided wave 14 cross-sectional weights that were estimated by integrating the initial sample weights and the top-up sample weights at the household level and the person level to ensure that weighted household and person estimates matched several known household-level and person-level benchmarks. This was followed by the incorporation of non-response adjustments [42].

Subsequently, linear regression analysis was conducted to examine the relationship between migrant status and job insecurity (as continuous outcomes). Then, gender, age, educational attainment, and occupational skill level were included in the linear regression one by one, and then simultaneously, to assess the potential for confounding [5,18]. However, educational attainment and occupational skill level showed a moderate correlation with each other (r = 0.49); thus, we conducted two separate ‘fully adjusted’ models, one including educational attainment and the other including occupational skill level. Following linear regression analyses, we conducted Likelihood ratio tests (LR tests) to assess whether gender, age, educational attainment, and occupational skill level moderated the association between migrant status and job insecurity. Interaction terms were fitted between migrant status and the potential moderator (gender, age, educational attainment, and occupational skill level) one by one. Where there was evidence of statistically significant effect modification, migrant status–job insecurity analyses were stratified by categories of the effect modifier. Finally, in the stratified analyses, the relationships between the significant effect modifier and job insecurity were tested for Australia-born workers and each subgroup of migrant workers, adjusting for potential confounders.

All analyses were conducted using Stata 15.1 (StataCorp LLC, College Station, TX, USA) [43].

## 3. Results

Table 1 shows the respondent characteristics without adjustment for sample weights. We included 9043 employed people in wave 14, among whom, 1749 (19%) were overseas-born workers. There were some notable differences in gender, age, educational attainment, and occupational skill level between Australia-born workers and migrant workers. For example, Australia-born workers included a significantly lower proportion of males (50% vs. 57%, *p* < 0.001) and workers in high-skill level jobs (37% vs. 46%, *p* < 0.001) compared to main-ESC-born workers. Further, Australia-born workers included a significantly lower proportion of workers with a postgraduate degree (12%) than overseas-born workers (22%, *p* < 0.001), which may be explained by Australia’s preference for skilled migrants. In contrast, Australia-born workers included a significantly higher proportion of workers between 15 to 24 years of age (21%) than overseas-born workers (6%, *p* < 0.001).

There were 1532 respondents (14% of the employed sample) who were missing one, two, or all three items in the job insecurity scale; these were excluded from our analysis. According to the reasons for missing data provided by HILDA, the vast majority (*n* = 1184) were due to participants not responding to the self-completion questionnaire (SCQ) of the HILDA survey, which is where the job insecurity questions were asked. Other reasons included not being asked (*n* = 286), multiple responses to the SCQ (*n* = 12), and refused/not stated (*n* = 50). Among the 50 who refused to answer the items, 9 were missing all three items, 2 were missing two items, and 39 were missing one item. Based on these reasons, a total of 1479 (1184 + 286 + 9) were missing all three items of the job insecurity scale, plus a small number (*n* = 12) were excluded due to multiple responses (ambiguous/invalid responses), leaving 41 respondents that were missing one or two items of the job insecurity scale. Compared to the observations that were included in our analysis, these exclusions were more likely to be male (57% vs. 51%, *p* < 0.001), younger (15–24 years, 24% vs. 18%, *p* < 0.001), lower educated (high school or lower, 39% vs. 34%, *p* < 0.001), lower in skill level (low-skill level, 18% vs. 14%; high-skill level, 33% vs. 39%, *p* < 0.001), and non-ESC-born migrants (14% vs. 10%, *p* < 0.001).

### 3.1. Are Exposures to Job Insecurity Different between Migrant Workers and Native Workers?

In both the unadjusted and fully adjusted models (Table 2), migrant workers, except main-ESC-born workers, showed significantly higher insecurity than Australia-born workers. The mean differences in job insecurity between Australia-born workers and migrant workers narrowed gradually with increasing years after arrival. Beyond 11 years post-arrival, the gap in the fully adjusted model was statistically indistinguishable from Australia-born workers; this was the case when controlling for either educational attainment or occupational skill level. Further, adjustment for gender, age, educational attainment, or occupational skill level separately did not substantially affect the results (see Appendix Table A1).

### 3.2. Is the Migrant Status-Job Insecurity Relationship Modified by Gender, Age, Educational Attainment, or Occupational Skill Level?

When effect modifiers were tested, the only significant interaction was found between educational attainment and migrant status based on COB (LR test results: COB × education: *p* = 0.04; dominant language of COB × education: *p* = 0.34; years since arrival × education: *p* = 0.25). There was no evidence of effect modification of the migrant status–job insecurity relationship by gender (LR test results: COB × gender: *p* = 0.42; dominant language of COB × gender: *p* = 0.83; years since arrival × gender: *p* = 0.70), age (LR test results: COB × age: *p* = 0.57; dominant language of COB × age: *p* = 0.63; years since arrival × age: *p* = 0.27), or occupational skill level (LR test results: COB × skill level: *p* = 0.30; dominant language of COB × skill level: *p* = 0.24; years since arrival × skill level: *p* = 0.38). The main effects and interaction effects of migrant status and educational attainment are shown in Appendix Table A2.

Table 3 presents three sets of results for the migrant status–job insecurity relationships stratified by level of educational attainment (migrant status was measured in three different ways). From the first measure of migrant status in the table, it can be seen that when compared to Australia-born workers, overseas-born workers with bachelor and postgraduate degrees had significantly higher job insecurity. In the second measure of migrant status, results show that this disparity was driven by non-ESC-born migrants. Finally, in the third measure of migrant status, it can be seen that migrants who were within their first five years post-arrival had the highest insecurity across all educational levels, but this disparity only persisted with increasing time since arrival for the most highly educated workers (bachelor and postgraduate degree holders).

For completeness, we also made within-group comparisons. In Australia-born workers, the job insecurity of those with diploma degrees was significantly higher (β = 0.25, *p* = 0.02), while job insecurity of those with a postgraduate degree was significantly lower (β = −0.36, *p* = 0.02), than the Australia-born workers with high school or lower education levels. Within the migrant worker group (including all subgroups), similar job insecurity levels were reported regardless of their educational level. The only exception occurred when migrant status was measured in terms of overseas-born, in which migrants with diploma degrees had significantly higher job insecurity (β = 0.59, *p* = 0.03) than migrants with high school or lower education levels.

## 4. Discussion

Consistent with our broad hypothesis, migrant workers, especially non-ESC-born and recently arrived migrants, experienced higher job insecurity than Australia-born workers. These disparities gradually decreased as years since arrival in Australia increased, becoming similar to Australia-born workers after 11+ years post-arrival, with a notable exception regarding highly educated migrants, for whom disparities persisted. Importantly, the migrant status–job insecurity relationships in our study were not confounded by gender, age, educational attainment, or occupational skill level. However, migrant status–job insecurity relationships were modified by educational attainment, and the disparities in job insecurity between non-ESC-born migrants and Australia-born workers increased with increasing educational attainment, contrary to expectation.

There are a limited number of previous studies directly comparing job insecurity between migrant workers and native workers, with most of them reporting that migrant workers experienced higher job insecurity [28,33,35,37]. For example, Kim-Godwin and Bechtel [33] showed that migrant farmworkers reported high job insecurity in the US, and Font, Moncada, Llorens, and Benavides [28] reported that migrant workers experienced higher job insecurity in Spain. Null results were also reported. An Australian study restricted to skilled migrants found no significant difference in job insecurity compared to Australia-born workers [36], and Torá et al. [44] also reported that migrant workers showed no significant difference in job insecurity compared to Spanish-born workers. In addition to differences between countries, inconsistencies in the results of our study compared to previous studies may have been due to differences in the samples, job stressor measurements, and analytical methods. For example, some studies looked at job insecurity in specific occupational groups, such as farmworkers, without comparison to reference groups [33]. Some studies compared migrants to native-born workers, but did not study migrant subgroups based on language proficiency or time since arrival in the host country [36,37]. Finally, job insecurity measures vary between studies. Job insecurity in McGuinness and Wooden [35] was measured by the percent chance of involuntary job loss, re-employment prospects, and intention to quit. Calculation methods for job insecurity may also have differed; some studies used binary job insecurity [37,44], while we treated job insecurity as a continuous measure.

With respect to research question one, our finding that the disparities in job insecurity were more significant among non-ESC-born migrants and decreased with increasing years post-arrival in Australia may indicate an acculturation effect. This may be explained by new migrants, especially those with low English proficiency, being more likely to find temporary or lower quality jobs [27], or having a higher risk of job loss due to their low language skills, unfamiliarity with local policies, or discrimination, which would correlate with lower job security. With respect to research question two, we found that only educational level was a significant effect modifier of migrant status–job insecurity relationships. Language difficulties may be more notable among newly arrived, low-educated migrants, and this may partly explain the result that migrant workers with high school or lower educational attainment had the highest levels of job insecurity in the first five years since arrival. Over time, these people may have improved chances of finding better jobs with better job security [45]. Further, we observed an unexpected finding that the disparities in job insecurity persisted among highly educated non-ESC-born migrants even 11+ years post-arrival, which may be partly explained by language difficulties having persistent adverse effects on work experience among highly educated migrant workers. Highly educated migrants were more likely to be employed in professional jobs, which require a high level of language skills [46]. Non-ESC-born migrants may have more language difficulties in a functional communication context, which may result in a perceived higher risk of future job loss. For example, language difficulty was regarded as the primary weakness of international medical residents in hospitals [47], which may result in a high risk of obtaining negative feedback and failure to register [48], thereby leading to increased job insecurity. Therefore, continuing language training or language training for a specific purpose may be beneficial to highly educated migrants [49].

Moreover, we found that higher educated migrant workers had similar job insecurity levels to lower educated migrants, which was inconsistent with previous working population-level results suggesting that higher levels of education are associated with greater job security [5,17]. This may indicate another possible contributor to high job insecurity among highly educated migrants, i.e., status inconsistency, which refers to “a discrepancy between the position a person holds in one domain of their social environment comparative to their position in another domain “ [50], including being overeducated for a job or having limited opportunities to use skills in a job [51]. In another study using the same survey data, we showed that highly educated migrant workers had lower skill discretion and complexity, which may indicate status inconsistency. Overeducated workers for their current jobs were reported to be more likely to have short job tenure [52], have lower job satisfaction [53], have higher levels of quit intention, and higher rates of job mobility [35], all of which could be associated with higher job insecurity. Further, workers who are overqualified or over-skilled may be recognized as relatively less proficient for the mismatched job, regardless of their higher educational attainment. From our results, migrant workers with high school or lower educational attainment had similar job insecurity to Australia-born workers 5+ years post-arrival, which may be partly because lower educated workers may have a lower risk of status inconsistency. Moreover, discrimination may also play a role in higher job insecurity among overeducated migrants, because job insecurity combined with ethnic discrimination has been reported to be the most common combination of psychosocial job stressors among Australian migrant workers [34]. Ethnic and racial minority groups, including migrants, have been reported to be targets of discrimination-related job loss [54], resulting in migrants being the ‘last hired’ and ‘first fired’ [55]. Therefore, if there is a risk of downsizing, migrant workers may have a higher risk of being fired compared to native workers, especially when they are recognized as less proficient.

Some limitations should be considered in the interpretation of our findings. First, we used a cross-sectional design, so we could not formally conclude that migrant status caused more adverse job stressor exposures. However, migrant status was a stable characteristic and preceded job stressor exposures, with little possibility of reverse causation. Another possible limitation is the healthy worker effect, wherein migrants who dropped out the workforce due to poor working conditions may have been missed due to the cross-sectional design, thereby resulting in underestimation of the job stressor exposures of migrant workers. Second, we measured only subjective job insecurity, which may have varied by individual or population groups. For example, Erlinghagen [45] reported that the perception of job insecurity had significant cross-country differences. Third, the variable of the dominant language of the country of birth may have included some migrant workers whose dominant language was not English, but they still spoke English proficiently; however, this exposure misclassification would only affect non-ESC-born workers and would bias our results towards the null (making it harder to observe differences). Fourth, discrimination against ethnic minorities may have played a vital role in migrants’ high job insecurity. Based on the consideration that non-ESC-born workers in HILDA included workers from ethnic minority groups, they may have been the target of discrimination in workplaces. A previous study using HILDA data also showed that migrant workers were more likely to report failure to get a job due to discrimination [56]. However, HILDA did not include direct measures of race by ethnicity, which precluded further analysis in this area. Fifth, most of the respondents with missing items for the job insecurity scale were due to non-response to the self-completion questionnaire (SCQ) of the HILDA survey, rather than missing individual items. Those who did not answer the SCQ were more likely to be male, younger, lower educated, working a low-skill level job, from a non-English speaking country, have lower English proficiency, have a disability, or have long-term disease [57]. These population groups were more likely to experience higher job insecurity [18], therefore, this most likely resulted in underestimation of job insecurity exposures. Sixth, a bilingual interview was available for only the most common non-English languages in Australia and only for a small number of cases; therefore, because the self-completion questionnaire was only provided in English [42], the results were likely to be biased towards an underestimation of migrant versus non-migrant differences.

This study has particular strengths. It presents a thorough comparison of job insecurity of migrants and non-migrants using a national population-representative sample. We also used a predictively validated measure of job insecurity [58], and the study unpacks the relationships between migrant status and job stressor exposures in detail by defining migrant status in three different ways. Finally, we systematically tested the modifying effects of gender, age, educational attainment, and occupational skill level on the associations of migrant status and job insecurity, revealing more nuanced relationships than previously reported.

## 5. Conclusions

In conclusion, non-ESC-born workers have significantly higher levels of job insecurity than Australia-born workers, especially for those with high educational attainment. Although the disparity in job insecurity between Australia-born workers and migrant workers decreases with the increasing number of years post-arrival in Australia, the difference is still significant among highly educated migrants even 11+ years post-arrival. Considering the associations between job insecurity and a wide range of adverse physical and mental health outcomes, the persisting disparities in job insecurity among highly educated migrant workers deserves further attention, and future research on its causes and its effect on migrants’ health and well-being is warranted. Our results also suggest that policies and practices to improve migrants’ English language ability and to prevent discrimination could reduce disparities in job insecurity and facilitate the integration of migrants into the Australian labour market.

## Figures and Tables

**Table 1:** Descriptive statistics on socio-demographic characteristics by migrant status (without weights, *n* = 9043).

Socio-Demographic Characteristics	Australia-Born, *n* (%)	Overseas-Born, *n* (%)	Main-ESC-Born ^†^, *n* (%)	Non-ESC-Born ^†^, *n* (%)	Arrived ≤5 Years Ago, *n* (%)	Arrived 6–10 Years Ago, *n* (%)	Arrived ≥11 Years Ago, *n* (%)
**Overall**	7291 (80.65)	1749 (19.35)	808 (8.94)	941 (10.41)	156 (1.73)	204 (2.26)	1388 (15.36)
**Gender**							
Male	3649 (50.05)	932 (53.29)	464 (57.43)	468 (49.73)	77 (49.36)	114 (55.88)	740 (53.31)
Female	3642 (49.95)	817 (46.71)	344 (42.57)	473 (50.27)	79 (50.64)	90 (44.12)	648 (46.69)
**Age (years)**							
15–24	1499 (20.56)	110 (6.29)	45 (5.57)	65 (6.91)	29 (18.59)	25 (12.25)	56 (4.03)
25–34	1673 (22.95)	376 (21.50)	135 (16.71)	241 (25.61)	86 (55.13)	84 (41.18)	206 (14.84)
35–44	1507 (20.67)	419 (23.96)	184 (22.77)	235 (24.97)	29 (18.59)	66 (32.35)	324 (23.34)
45–54	1591 (21.82)	496 (28.36)	270 (33.42)	226 (24.02)	8 (5.13)	25 (12.25)	463 (33.36)
55–64	1021 (14.00)	348 (19.90)	174 (21.53)	174 (18.49)	4 (2.56)	4 (1.96)	339 (24.42)
**Educational attainment**							
High school or lower	2647 (36.31)	401 (22.93)	218 (26.98)	183 (19.45)	28 (17.95)	39 (19.12)	335 (24.14)
Diploma or certificate	2550 (34.98)	563 (32.19)	304 (37.62)	259 (27.52)	39 (25.00)	46 (22.55)	476 (34.29)
Bachelor	1235 (16.94)	408 (23.33)	143 (17.70)	265 (28.16)	51 (32.69)	59 (28.92)	298 (21.47)
Postgraduate	858 (11.77)	377 (21.56)	143 (17.70)	234 (24.87)	38 (24.36)	60 (29.41)	279 (20.10)
**Occupational skill level**							
Lowest level	1027 (14.10)	241 (13.78)	103 (12.75)	138 (14.67)	28 (17.95)	27 (13.24)	185 (13.33)
Mid–low level	1703 (23.38)	326 (18.64)	136 (16.83)	190 (20.19)	35 (22.44)	36 (17.65)	255 (18.73)
Mid–high level	1851 (25.42)	405 (23.16)	201 (24.88)	204 (21.68)	41 (26.28)	45 (22.06)	319 (22.98)
Highest level	2702 (37.10)	777 (44.43)	368 (45.54)	409 (43.46)	52 (33.33)	96 (47.06)	629 (45.32)

^†^ Main-ESC-born: Born in a main-English speaking country; non-ESC-born: Born in a non-English speaking country. * Summations of percentage for some columns were not equal to 100% due to rounding.

**Table 2:** Weighted mean and unadjusted and adjusted mean differences in job insecurity between Australia-born and migrant workers (*n* = 9043).

Migrant Status		Mean (SE^®^)	Unadjusted		Adjusted ^‡^ (Educational Attainment)		Adjusted ^∆^ (Occupational Skill Level)	
			Coefficient	95% CI	Coefficient	95% CI	Coefficient	95% CI
Exposure 1: Based on COB ^ѱ^	Australia-born	8.49 (0.06)	--		--		--	
	Overseas-born	9.09 (0.15)	0.45 ***	0.25; 0.65	0.44 ***	0.24; 0.65	0.42 ***	0.22; 0.62
Exposure 2: Based on COB ^ѱ^ and dominant language of COB	Australia-born	8.49 (0.06)	--		--		--	
	Main-ESC-born ^†^	8.96 (0.20)	0.18	-0.10; 0.46	0.10	−0.18; 0.38	0.11	−0.18; 0.39
	Non-ESC-born ^†^	9.19 (0.18)	0.69 ***	0.42; 0.95	0.74 ***	0.48; 1.01	0.68 ***	0.42; 0.95
Exposure 3: Based on COB ^ѱ^ and years since arrival in Australia	Australia-born	8.49 (0.06)	--		--		--	
	Arrived ≤5 years ago	10.03 (0.37)	1.52 ***	0.90; 2.13	1.76 ***	1.14; 2.38	1.66 ***	1.05; 2.27
	Arrived 6–10 years ago	9.47 (0.30)	0.83 **	0.29; 1.37	0.96 ***	0.42; 1.50	0.90 **	0.36; 1.43
	Arrived ≥11 years ago	8.79 (0.17)	0.26 *	0.04; 0.49	0.20	−0.03; 0.43	0.19	−0.04; 0.41

COB ^ѱ^: Country of birth; ^†^ main-ESC-born: Born in a main-English speaking country; non-ESC-born: Born in a non-English speaking country; SE**^®^**: Standard error was calculated after weights. Household Income and Labour Dynamics in Australia Survey (HILDA) User Manual-Release 14 (Summerfield et al. 2015) provided wave 14 cross-sectional weights that were used for data users; **^‡^** This fully adjusted model adjusted for gender, age, and educational attainment, simultaneously; ^∆^ this fully adjusted model adjusted for gender, age, and occupational skill level, simultaneously. * *p* < 0.05, ** *p* < 0.01, *** *p* < 0.001.

**Table 3:** Mean differences in job insecurity between Australia-born workers and migrant workers by educational attainment, adjusted for gender and age (*n* = 9043).

Migrant Status		High School or Lower		Diploma or Certificate		Bachelor		Postgraduate	
		Coefficient	95% CI	Coefficient	95% CI	Coefficient	95% CI	Coefficient	95% CI
Exposure 1: Based on COB ^ѱ^	Australia-born	--		--		--		--	
	Overseas-born	0.06	−0.34; 0.46	0.29	−0.07; 0.65	0.79 ***	0.36; 1.23	0.80 ***	0.34; 1.27
Exposure 2: Based on COB ^ѱ^ and dominant language of COB	Australia-born	--		--		--		--	
	Main-ESC-born ^†^	−0.21	−0.74; 0.32	0.12	−0.36; 0.59	0.33	−0.35; 1.01	0.36	−0.32; 1.05
	Non-ESC-born ^†^	0.38	−0.19; 0.95	0.49	−0.01; 0.99	1.04 ***	0.52; 1.56	1.08 ***	0.51; 1.64
Exposure 3: Based on COB ^ѱ^ and years since arrival in Australia	Australia-born	--		--		--		--	
	Arrival ≤5 years	2.38 **	0.98; 3.78	1.56 *	0.32; 2.81	1.77 **	0.67; 2.87	1.58 *	0.30; 2.85
	Arrival 6–10 years	0.84	−0.35; 2.03	1.15 *	0.01; 2.29	0.69	−0.33; 1.71	1.35 *	0.32; 2.38
	Arrival ≥11 years	−0.26	−0.69; 0.18	0.07	−0.32; 0.46	0.64 *	0.13; 1.14	0.59 *	0.06; 1.11

COB ^ѱ^: Country of birth; ^†^ Main-ESC-born: Born in a main-English speaking country; non-ESC-born: Born in a non-English speaking country. * *p* < 0.05, ** *p* < 0.01, *** *p* < 0.001.

## Appendix

**Table A1:** Mean differences in job insecurity between Australia-born workers and migrant workers, unadjusted and adjusted for gender, age, education attainment, and occupational skill level, separately (*n* = 9043).

Migrant Status		Unadjusted		Adjusted for Gender		Adjusted for Age		Adjusted for Education		Adjusted for Skill Level	
		Coeff	95% CI	Coeff	95% CI	Coeff	95% CI	Coeff	95% CI	Coeff	95% CI
**Exposure 1: Based on COB ^ѱ^**	Australia-born	--		--		--		--		--	
	Overseas-born	0.45 ***	0.25; 0.66	0.44 ***	0.24; 0.64	0.42 ***	0.22; 0.63	0.49 ***	0.28; 0.69	0.47 ***	0.26; 0.67
**Exposure 2: Based on COB ^ѱ^ and dominant language of COB**	Australia-born	--		--		--		--		--	
	Main-ESC-born ^†^	0.18	-0.10; 0.46	0.14	−0.14; 0.42	0.12	−0.16; 0.41	0.18	−0.10; 0.46	0.20	−0.08; 0.48
	Non-ESC-born ^†^	0.69 ***	0.43; 0.95	0.69 ***	0.43; 0.96	0.68 ***	0.42; 0.95	0.75 ***	0.49; 1.02	0.70 ***	0.43; 0.96
**Exposure 3: Based on COB ^ѱ^ and years since arrival in Australia**	Australia-born	--		--		--		--		--	
	Arrived ≤5 years	1.52 ***	0.90; 2.13	1.52 ***	0.91; 2.13	1.69 ***	1.07; 2.31	1.60 ***	0.98; 2.21	1.48 ***	0.87; 2.10
	Arrived 6–10 years	0.86 **	0.32; 1.40	0.80 **	0.27; 1.34	0.90 **	0.36; 1.44	0.93 **	0.39; 1.47	0.86 **	0.32; 1.39
	Arrived ≥11 years	0.26 *	0.04; 0.49	0.25 *	0.03; 0.47	0.19	−0.04; 0.41	0.29 *	0.07; 0.51	0.28 *	0.06; 0.51

COB ^ѱ^: Country of birth; ^†^ Main-ESC-born: Born in a main-English speaking country; non-ESC-born: Born in a non-English speaking country. * *p* < 0.05, ** *p* < 0.01, *** *p* < 0.001.

**Table A2:** The main effects and interaction effects of migrant status and educational attainment of the relationships between migrant status and job insecurity, adjusted for gender and age (*n* = 9043).

Migrant Status	Main Effects and Interaction Effects (Migrant Status × Educational Attainment)		Job Insecurity		
			Coefficients	*p* Value	95% CI
**Exposure 1:** **Based on COB ^ѱ^**	Main effects	Australia-born	--		
		Overseas-born	0.04	0.83	−0.37; 0.45
		High school or lower	--		
		Diploma	0.28	0.01	0.06; 0.50
		Bachelor	-0.19	0.15	−0.46; 0.07
		Postgraduate	-0.35	0.03	−0.65; −0.04
	Interaction effects	Australia-born × high school or lower	--		
		Overseas-born × diploma	0.24	0.38	−0.30; 0.78
		Overseas-born × bachelor	0.71	0.02	0.12; 1.30
		Overseas-born × postgraduate	0.74	0.02	0.12; 1.36
**Exposure 2:** **Based on COB ^ѱ^ and dominant language of COB**	Main effects	Australia-born	--		
		Main-ESC-born ^†^	−0.25	0.36	−0.79; 0.28
		Non-ESC-born ^†^	0.39	0.19	−0.19; 0.98
		High school or lower	--		
		Diploma	0.28	0.01	0.06; 0.49
		Bachelor	−0.19	0.16	−0.46; 0.07
		Postgraduate	−0.35	0.03	−0.65; −0.04
	Interaction effects	Australia-born × high school or lower	--		
		Main-ESC-born ^†^ × diploma	0.39	0.27	−0.31; 1.10
		Main-ESC-born ^†^ × bachelor	0.54	0.22	−0.32; 1.39
		Main-ESC-born ^†^ × postgraduate	0.60	0.18	−0.27; 1.47
		Non-ESC-born ^†^ × diploma	0.06	0.88	−0.71; 0.82
		Non-ESC-born ^†^ × bachelor	0.61	0.12	−0.16; 1.39
		Non-ESC-born ^†^ × postgraduate	0.65	0.11	−0.15; 1.46
**Exposure 3:** **Based on COB ^ѱ^ and years since arrival in Australia**	Main effects	Australia-born	--		
		Arrived ≤5 years ago	2.38	0.001	0.95; 3.81
		Arrived 6–10 years ago	0.83	0.18	−0.38; 2.05
		Arrived ≥11 years ago	−0.27	0.23	−0.72; 0.17
		High school or lower	--		
		Diploma	0.27	0.02	0.05; 0.49
		Bachelor	−0.20	0.15	−0.46; 0.07
		Postgraduate	−0.36	0.02	−0.67; −0.06
	Interaction effects	Australia-born × high school or lower	--		
		Arrived ≤5 years ago × diploma	−0.98	0.31	−2.87; 0.91
		Arrived ≤5 years ago × bachelor	−0.54	0.55	−2.34; 1.25
		Arrived ≤5 years ago × postgraduate	−0.75	0.44	−2.66; 1.15
		Arrived 6–10 years ago × diploma	0.26	0.76	−1.42; 1.93
		Arrived 6–10 years ago × bachelor	−0.25	0.76	−1.83; 1.33
		Arrived 6–10 years ago × postgraduate	0.45	0.58	−1.13; 2.03
		Arrived ≥11 years ago × diploma	0.35	0.23	−0.23; 0.94
		Arrived ≥11 years ago × bachelor	0.86	0.01	0.21; 1.52
		Arrived ≥11 years ago × postgraduate	0.83	0.02	0.15; 1.51

COB ^ѱ^: Country of birth; ^†^ Main-ESC-born: Born in main-English speaking country; non-ESC-born: Born in non-English speaking country; × means interaction.
